# Neonatal resuscitation and immediate newborn assessment and stimulation for the prevention of neonatal deaths: a systematic review, meta-analysis and Delphi estimation of mortality effect

**DOI:** 10.1186/1471-2458-11-S3-S12

**Published:** 2011-04-13

**Authors:** Anne CC Lee, Simon Cousens, Stephen N Wall, Susan Niermeyer, Gary L Darmstadt, Waldemar A Carlo, William J Keenan, Zulfiqar A Bhutta, Christopher Gill, Joy E Lawn

**Affiliations:** 1 Department of International Health, Johns Hopkins Bloomberg School of Public Health, Baltimore, MD, USA; 2 Department of Newborn Medicine, Brigham and Women’s Hospital, Boston, MA, USA; 3 London School of Tropical Medicine and Hygiene, London, UK; 4 Saving Newborn Lives/Save the Children; 5 Department of Pediatrics, Section of Nenoatology, University of Colorado, Aurora, CO, USA; 6 Integrated Health Solutions Development, Global Health Program, Bill & Melinda Gates Foundation, Seattle, WA, USA; 7 Department of Pediatrics, Division of Neonatology, University of Alabama at Birmingham, AL, USA; 8 Division of Neonatology, St. Louis University, St. Louis, MO, USA; 9 Division of Women & Child Health, the Aga Khan University, Karachi, Pakistan; 10 Department of International Health, Boston University School of Public Health, Boston, USA

## Abstract

**Background:**

Of 136 million babies born annually, around 10 million require assistance to breathe. Each year 814,000 neonatal deaths result from intrapartum-related events in term babies (previously “birth asphyxia”) and 1.03 million from complications of prematurity. No systematic assessment of mortality reduction from tactile stimulation or resuscitation has been published.

**Objective:**

To estimate the mortality effect of immediate newborn assessment and stimulation, and basic resuscitation on neonatal deaths due to term intrapartum-related events or preterm birth, for facility and home births.

**Methods:**

We conducted systematic reviews for studies reporting relevant mortality or morbidity outcomes. Evidence was assessed using GRADE criteria adapted to provide a systematic approach to mortality effect estimates for the Lives Saved Tool (LiST). Meta-analysis was performed if appropriate. For interventions with low quality evidence but strong recommendation for implementation, a Delphi panel was convened to estimate effect size.

**Results:**

We identified 24 studies of neonatal resuscitation reporting mortality outcomes (20 observational, 2 quasi-experimental, 2 cluster randomized controlled trials), but none of immediate newborn assessment and stimulation alone. A meta-analysis of three facility-based studies examined the effect of resuscitation training on intrapartum-related neonatal deaths (RR= 0.70, 95%CI 0.59-0.84); this estimate was used for the effect of facility-based basic neonatal resuscitation (additional to stimulation). The evidence for preterm mortality effect was low quality and thus expert opinion was sought. In community-based studies, resuscitation training was part of packages with multiple concurrent interventions, and/or studies did not distinguish term intrapartum-related from preterm deaths, hence no meta-analysis was conducted. Our Delphi panel of 18 experts estimated that immediate newborn assessment and stimulation would reduce both intrapartum-related and preterm deaths by 10%, facility-based resuscitation would prevent a further 10% of preterm deaths, and community-based resuscitation would prevent further 20% of intrapartum-related and 5% of preterm deaths.

**Conclusion:**

Neonatal resuscitation training in facilities reduces term intrapartum-related deaths by 30%. Yet, coverage of this intervention remains low in countries where most neonatal deaths occur and is a missed opportunity to save lives. Expert opinion supports smaller effects of neonatal resuscitation on preterm mortality in facilities and of basic resuscitation and newborn assessment and stimulation at community level. Further evaluation is required for impact, cost and implementation strategies in various contexts.

**Funding:**

This work was supported by the Bill & Melinda Gates Foundation through a grant to the US Fund for UNICEF, and to the Saving Newborn Lives program of Save the Children, through Save the Children US.

## Background

Initiation of breathing is critical in the physiologic transition from intra-uterine to extra-uterine life. Between 5-10% of all newborns require assistance to establish breathing at birth [1-6], and simple warming, drying, stimulation and resuscitation may reduce neonatal mortality and morbidity. Each year an estimated 814,000 neonatal deaths [8] are related to intrapartum hypoxic events in term infants, previously termed “birth asphyxia” [7], and over one intrapartum million stillbirths occur. Especially in under-resourced settings it may be challenging to distinguish a stillborn from a severely depressed newborn. In addition over one million newborns die from complications of preterm birth, such as respiratory distress syndrome [10], and these babies also require assistance to breathe at birth.

**Figure 1:**
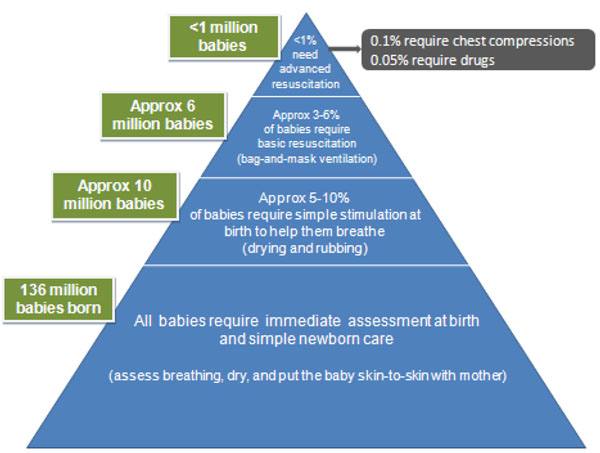
Estimate of annual number of all newborns who require assistance to breathe at birth and varying levels of neonatal resuscitation. Legend: Adapted from [1] using data from [2,3,5,6,20]

Neonatal resuscitation is defined as the set of interventions at the time of birth to support the establishment of breathing and circulation [6]. Of 136 million births annually, an estimated 10 million will require some level of intervention [1]. Some non-breathing babies with primary apnea will respond to simple stimulation alone, such as drying and rubbing (Figure 1). Basic resuscitation with a bag-and-mask is required for an estimated 6 million of these babies each year, and is sufficient to resuscitate most neonates with secondary apnea, as their bradycardia primarily results from hypoxemia and respiratory failure[6]. More advanced measures, including endotracheal intubation, chest compressions and medications are required in <1% of births (Figure 1) [3,11], and most of these babies require ongoing intensive care which is not available in most low income country settings. Supplemental oxygen is not associated with survival benefit in term infants [12], although the effect may differ in very preterm infants [13-15].

While systematic training in resuscitation of the newborn is a cornerstone of modern neonatology, there have been few rigorous evaluations of its effectiveness, partly because the intervention was standard practice before the advent of randomized controlled trials (RCTs), and randomization of individuals or clusters to no treatment would now be considered unethical. However, in low income countries, particularly in South Asia and sub-Saharan Africa, which account for over two-thirds of the world’s neonatal deaths [10], resuscitation is not available for the majority of newborns who are born either at poorly staffed and equipped first-level health facilities, or at home (60 million births annually), where birth attendants may lack skills or may perform practices that delay effective ventilation [1].

Neonatal resuscitation is receiving increasing attention especially as a missed opportunity for saving lives for births already in facilities, and for improving morbidity outcomes. Increased momentum for scale up in low-middle income countries has resulted from the release of a simplified resuscitation algorithm and training package led by the American Academy of Pediatrics (http://www.helpingbabiesbreathe.org/), evidence that neonatal resuscitation with room air is effective, and the invention of lower cost, appropriate equipment and training manikins, plus a consortium of implementing partners. In a survey of policymakers and programme managers regarding “birth asphyxia”, evaluating the effectiveness of neonatal resuscitation, particularly at the community level, emerged as a top research priority [19]. Several recent reviews of neonatal resuscitation in low-middle income settings [1,16-18] have concluded that neonatal resuscitation has the potential to save newborn lives; yet, effect estimates of mortality reduction are lacking to guide program planners as to how many lives could be saved by immediate assessment and stimulation, which may be feasible with less skilled workers and no equipment, and the additional effect of basic neonatal resuscitation, including airway positioning and clearing, and bag-mask resuscitation [20] (table 1).

**Table 1:** Definition of Interventions


**Immediate assessment and stimulation of the newborn baby**
Immediate assessment, warming, drying and tactile stimulation (rubbing with the drying cloth, rubbing the back or flicking the feet) of the newborn at the time of birth. This is not the same as the WHO package of essential newborn care which is more complex and includes immediate breastfeeding, resuscitation, thermal care, eye care, immunization etc.

**Basic Newborn Resuscitation**
Airway clearing (suctioning if required) head positioning and positive pressure ventilation via bag-and- mask.*

**Advanced Newborn Resuscitation** (not estimated for LiST)
Basic neonatal resuscitation (as above) *plus* endotracheal intubation, supplemental oxygen, chest compressions, and medications.

*Note: While basic newborn resuscitation includes immediate assessment and stimulation, *the effect estimated for the purposes of the LiST tool is the additional effect of basic resuscitation in addition to stimulation as the program implications differ in terms of skills and equipment.*

## Objective

The objective of this review is to provide estimates for use in the Lives Saved Tool (LiST), of the effect of immediate newborn assessment and stimulation, and the additional effect of basic neonatal resuscitation, on neonatal mortality from two causes of neonatal death (intrapartum-related deaths in term infants (“birth asphyxia”) and complications of preterm birth) and in two contexts (facility and community).

## Methods

This review is one of a series of standard reviews to provide consistent and transparent estimates of mortality effect used in the Lives Saved Tool (LiST), a model to assist evidence-based program planning. LiST is described in greater detail elsewhere [21]. In LiST, the estimation of lives saved depends on national estimates of causes of death for mothers, newborns and children under five, and the planned changes in national coverage estimates for given interventions, with a resultant reduction in cause-specific mortality. The sources and methods for each input are being provided in the public domain. The cause of death data is developed by the Child Health Epidemiology Reference Group (CHERG) with the United Nations each year and includes a country review process [8]. Intervention coverage data is based on national coverage estimates, or in the absence of appropriate recent data, the assumptions are described elsewhere [22,23]. This mortality effect review follows standard methods adapted from GRADE [24] by the CHERG as described previously [21].

### Searches

We undertook a systematic review of the literature from 1980 until March 2010. The following databases were searched without language restrictions but limited to “human ”: PubMed, Popline, Cochrane, EMBASE, IMEMR (Index Medicus for the WHO Eastern Mediterranean Region), LILACS (Latin American and Caribbean Health Sciences Literature), and African Index Medicus. The search terms included MeSH terms and combinations of “newborn/neonatal resuscitation,” “neonatal mortality,” “birth asphyxia,” and “asphyxia neonatorum.” Snowball searching added literature referenced in key papers. The review for immediate newborn assessment and stimulation was conducted as part of extensive literature reviews of interventions for “birth asphyxia” [7]. Efforts were also made to contact investigators and program managers for unpublished data.

### Inclusion/exclusion criteria for abstraction

Data from studies meeting the inclusion criteria were extracted using a standard form and re-checked. We abstracted information on study identifiers, context, design and limitations, intervention definitions, and outcomes (table 1). We assessed the quality of each study using the standard approach adapted from GRADE [24] developed by the CHERG [21]. For studies with data missing or requiring clarification, we contacted principal investigators.

We used the PICO format for inclusion/exclusion – Patient, Intervention, Comparison, Outcome. The patient of interest is the newborn baby who is not breathing at birth. We considered the following study designs: randomized controlled trials, observational before-and-after or quasi-experimental. Only studies reporting outcomes for an intervention and a comparison or control group (either historical or concurrent) were included.

### Interventions definitions

We estimate the effects of two interventions:

1) *Immediate newborn assessment and stimulation* (warming, drying and rubbing the back or flicking soles of the feet).

2) *Basic newborn resuscitation*, defined as airway clearing (suctioning), head positioning and positive pressure ventilation via bag-and-mask or tube-and-mask (noting that tube-and-mask device is no longer recommended for use) (table 1)

While basic newborn resuscitation includes newborn assessment and stimulation, for the purposes of the LiST model, the estimate is of the additional incremental mortality effect. Advanced resuscitation procedures (including chest compressions, supplemental oxygen, intubation or administration of medications) are very rarely required (Figure 1), unfeasible or unavailable in most low-resource settings, and unlikely to have substantial additional mortality benefit over basic resuscitation in settings without ongoing neonatal intensive care. Thus, the aim of this review was to estimate the impact of basic resuscitation. We do not separately estimate the incremental mortality effect for advanced resuscitation procedures. The effect of breastfeeding, postnatal thermal care practices, and kangaroo mother care for preterm babies, are reviewed elsewhere for LiST and not included here.

### Outcomes definitions

A neonatal death was defined as a death in the first 28 days of life, early neonatal death as death in the first 7 days of life, and perinatal death as a stillbirth (≥1000 gms, ≥ 28 weeks gestation) or death in the first 7 days of life. Studies that reported neonatal mortality, early neonatal mortality, perinatal mortality, “asphyxia”-specific mortality, mortality from complications of preterm birth, or incidence of neonatal encephalopathy were included for assessment.

The definitions used for cause-specific neonatal mortality have changed over time. WHO has previously defined “birth asphyxia” as “the failure to initiate and sustain breathing at birth [20],” indicating the clinical need for neonatal resuscitation, a syndromic state also commonly referred to as neonatal or perinatal respiratory depression. This clinical approach combines two cause-specific mortality outcomes which should be separated for cause of death attribution, notably (1) term babies with intrapartum brain injury and (2) preterm infants who do not breathe at birth. The term “birth asphyxia” is no longer recommended for epidemiological use [25-27], especially for cause-of-death attribution, as it combines differing ICD categories with differing prevention strategies. The preferred terminology is “intrapartum-related neonatal death” which refers to term babies with neonatal encephalopathy, or death prior to onset of neonatal encephalopathy, and evidence of intrapartum injury or acute intrapartum events [9,26,28-30]. Preterm neonatal deaths have been defined by the CHERG based on ICD guidelines for as those deaths due to complications of preterm birth, including respiratory distress syndrome, intraventricular hemorrhage, and necrotizing enterocolitis, or with gestational age <34 weeks, or birth weight <2000 g [29].

We did not examine Apgar score as an outcome since our goal was to establish mortality effect estimates, and Apgar scores are an unreliable indicator of mortality, long term morbidity or cause (influenced by physiologic immaturity, infection, and medications during labour-delivery) [27,31].

### Analyses and summary measures

We conducted meta-analyses for mortality outcomes of observational before-and-after studies of neonatal resuscitation training in facility settings. Statistical analyses were performed using STATA 11. The Mantel-Haenszel pooled risk ratio (RR) or, when there was evidence of heterogeneity (p<0.10), the DerSimonian-Laird pooled risk ratio, was estimated together with a 95% confidence interval (CI). We summarized the overall quality of evidence for each outcome and each data input type using an adapted version of the GRADE protocol table [21,24].

### Delphi process for establishing expert consensus

For intervention-outcome combinations without moderate or high quality evidence, but with a strong GRADE recommendation for implementation, we sought expert opinion via a Delphi process [32]. We invited a panel of experts in newborn and public health including multiple disciplines – program management, research and clinical general pediatrics and neonatology. The questionnaire was developed by JL, SW, and ACL, and refined by pilot testing. The questionnaire was sent by email and included background to the Delphi process and asked for estimates of the effect for five scenarios (See Additional File 2). Respondents were allowed the option of anonymous response. Consensus was defined *a priori* as an inter-quartile range of responses to a given question of ≤ 30%.

## Results

**Figure 2:**
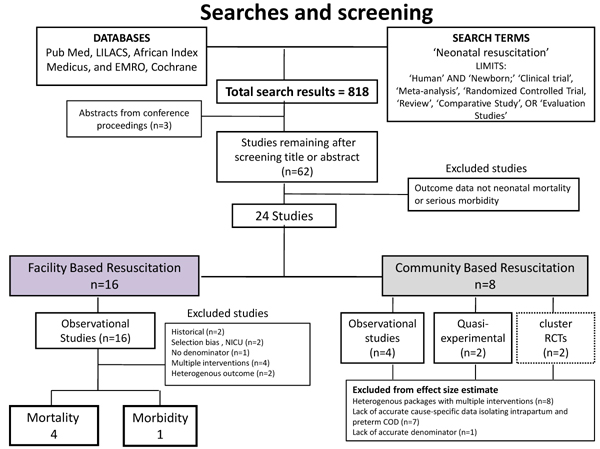
Search, screening and selection of studies reporting effect of neonatal resuscitation on neonatal mortality and morbidity

In the literature review, we identified 818 titles of articles of potential interest (Figure 2), and after initial screening of titles and abstracts, we retrieved 62 papers, reports or conference abstracts for review. We located 24 studies that reported the impact of neonatal resuscitation training on mortality outcomes: 16 studies in facilities, and 8 studies in community settings. Conference abstracts for 3 studies were identified and authors were contacted for further data, and there was one unpublished program report. All studies except one were from low or middle income settings. No studies were identified that examined the effect of newborn assessment and stimulation alone. The details of the studies are given in tables 2, 3 and 6.

The Delphi panel included eighteen experts (90% response rate) representing five WHO regions [Americas (n=6); Southeast Asia (n=4); Eastern Mediterranean (n=2); Africa (n=4); Europe (n=2)], from the following specialties: neonatology (n=7); general pediatrics (n=11) and pediatric infectious disease (n=1). Expert opinion was requested for 5 mortality effects (see additional file 2): facility- based basic resuscitation on preterm mortality, community-based basic resuscitation and immediate newborn assessment and stimulation on both intrapartum-related and preterm mortality. Consensus was reached in the first round for all 5 estimates.

## **Evidence for mortality impact of neonatal resuscitation training in facilities**

Of 16 observational, facility-based studies of neonatal resuscitation, 14 were before-after studies and 2 were historical reports. Details of each study and the main results are shown in Tables 2 and 3 and the assessment of quality of evidence according to GRADE is shown in table 4.

**Table 2:** Observational studies of neonatal resuscitation training programs in facility settings with mortality outcomes

Author	Setting/Country	Study Design	Intervention definition	Outcomes: definition	Distinguish Preterm from Intrapartum Deaths	N (Births) A = Baseline B = Endline	Effect Size RR/OR (95%CI)
Zhu XY et al 1997[3]	Urban Hospital China	Before-and-after study	AAP NRP training at of all delivery room staff at hospital	1) Early Neonatal Mortality (first 7 days): ALL cause	Not stated	A) 1,722; B) 4,751	1) RR 0.34 (0.17-0.67)

Deorari AK et al 2001[2]	14 University Hospitals, India	Before-and-after study	AAP NRP training of 2 faculty/hospital, subsequent training of DR room nurses and doctors; competency based certification	1) Asphyxia neonatal mortality [Features of fetal hypoxia and 5 min Apgar <6 following complications of pregnancy or delivery]; 2) Hypoxic Ischemic Encephalopathy; 3) Preterm mortality [BW < 1000 g with HMD, IVH or AOP]	Excluded BW < 1000 g, death from HMD/IVH or AOP	A) 7,070; B)25,713	1) RR 0.70 (0.56-0.87) 2) RR 1.68 (1.06-2.67) 3) RR 0.95 (0.74-1.24)

Vakrilova L et al 2005[44]	All hospitals with delivery rooms in Bulgaria	Before-and-after study	French-Bulgarian Program on Newborn Resuscitation, training in all obstetric wards in country	1) Asphyxia Neonatal Mortality [ICD 9 'perinatal and intrapartum asphyxia'], 2) Early neonatal mortality (first 7 days) 3) Preterm complication [ICD-9 'immaturity related' and 'respiratory distress syndrome']	Excluded death due to preterm complications by ICD-9	A) 67,948; B) 67,647	1) RR 0.83 (0.54-1.27) 2) RR 0.86 (0.74-1.01) 3) RR 1.33 (1.03-1.73)

Carlo, et all 2010 [38]/Chomba E et al 2008[39]	18 Urban Low-risk delivery centers, Zambia	Before-and-after study,then RCT	WHO ENC Package, including basic resuscitation with bag-mask,taught by demonstration, clinical practice sessions, and performance evaluations; followed by longer in depth training in NRP including bag-mask ventilation and chest compressions	1) Asphyxia Early Neonatal Mortality (7 d), [not breathing at birth]; 2) Early Neonatal Mortality [first 7 days]; 3) Preterm Mortality [preterm or BW <1500]	Preterm or LBW (< 1500 g) as separate cause of death, though no hierarchy specified for single cause of death	A) 8,148; B) 20,534	1) RR 0.56 (NS) 2) RR 0.60 (0.48-0.76) 3) RR 0.74 (NS)

**Table 3:** Additional observational studies of neonatal resuscitation training programs in facilities, excluded from meta-analysis

Author	Setting/Country	Study Design	Intervention definition	Outcomes: definition	Preterm vs. Intrapartum	N (Births) A = Baseline B = Endline	Effect Size RR/OR (95%CI)
Zhu et al* 1993[45]	Health center, Yinshan, China	Before-and-after study	ABCDE protocol of modern resuscitation with labour ward personel	1) Asphyxia Case Fatality	Not Stated	A) Number of resuscitations 184 223	1) RR 0.94

Tholpadi SR et al* 2000[40]	32 peripheral health centers; Kerala, India	Before-and-after study	AAP NRP Training of village health center physicians, nurses, birth attendants; performance checklist; refresher in 3 months	1) Asphyxia 2) Asphyxia Mortality (definitions not stated)	Not Stated	A) 874; B) 960	1) RR 0.68 (0.15-3.04)

Jeffery HE et al* 2004[33]	3 Tertiary care, 13 District Hospitals; Macedonia	Before-and-after study	10 month perinatal training program doctors and nurses (Neonatal resuscitation, thermal care, jaundice, respiratory distress syndrome, infection control)	1) PMR 2) Fetal mortality 3) NMR	< 1000 g excluded	A) 68,755 B) 44,263	1) RR 0.72 (0.66-0.78) 2) RR 0.79 (0.71-0.89) 3) RR 0.64 (0.56-0.72)

O'Hare BA et al* 2006[49]	Teaching Hospital; Kampala, Uganda	Before-and-after study	Team of nurses trained in basic resuscitation to attend all deliveries in 1 month period, performance based evaluation;	1) Mortality of NICU admissions	Preterms excluded	A) 1296; B) 1,046	20.8% in control vs. 17.3% in pilot

Duran R et al* 1998[42]	Tertiary Care Hospital; Trakya, Turkey	Before-and-after study	NRP courses in Trakya region, Turkey 2003 & 2004	1) "Asphyxia" NICU admissions 2) Duration of asphyxia hospitalization	Not Stated	Not Stated	1) 35 vs 13 NICU admissions for asphyxia 2) 15 to 6 days

Draycott et al* 2006[37]	Maternity Unit; South Meade, UK	Before-and-after study	EOC training course: CTG obstetric emergency drills, and neonatal resuscitation	1) HIE (MacLennan):	Not Stated	A) 8,430 B) 11,030	1) RR 0.50 (0.26-0.95)

Wang H et al* 2008[41]	17 general, 23 maternal child health hospitals; China	Before-and-after study	Nationwide AAP NRP training, started in 2004 in 20 provinces	1) Asphyxia Mortality [Delivery room death infant 1 min Apgar <7]	Preterms not excluded	A) 51,306; B) 68, 247	1) RR 0.67 (0.34-1.30)

Mufti P et al* 2006[35]	Teaching Hospital, Karachi, Pakistan	Before-and-after study	Training in management of low birthweight, respiratory distress, feeding, neonatal sepsis, and neonatal resuscitation.	1) PMR 2) NMR	Not Stated	A) 2871 B) 4106	1) RR 0.85 (0.69-1.05) 2) RR 0.72 (0.51-1.02)

Boo et al* 2009[43]	National training in all states Malaysia	Historical/ecological study	AAP NRP, national training and certification Perinatal Society; written/practical test for certification; retraining	1) PMR; 2) NMR (all cause)	Not Stated	National annual births over 8 years	Annual NMR reported over 8 years

Sen et al* 2009[34]	District Hospital, Purulia India	Before-and-after study	Training in neonatal resuscitation, equipping labor room-OR with resuscitation equipment.	1) Labor room death (hospital)	Not Stated	A) 5077 B) 6704	1) RR 0.56 (0.42-0.75)

Opiyo N et al* 2008[46]	Public Hospital, Nairobi, Kenya	RCT and before-after	Training of delivery room nurses-midwives in adapted UK resuscitation council. Written-clinical competency assessment.	1) NMR (all cause)	Not Stated	A) 4367 B) 4084	NMR 25(pre) vs 26.2 (post-intervention)

Berglund et al* 2010[36]	Three maternity wards, Ukraine	Before-and-after study	Training maternity staff WHO "Effective Perinatal Care" including emergency obstetric and neonatal care. All maternities equipped for resuscitation	1) Early NMR	Not Stated	A) 1696 B) 2439	No significant effect on ENMR

**Table 4:** GRADE assessment of studies of the effect of Neonatal Resuscitation training in facilities on neonatal mortality from Intrapartum-related events (ie. “birth asphyxia”)

No of studies	Design	Limitations	Consistency	Generalizability to Population of Interest	Generalizability of intervention of interest	**Post-InterventionEvents**	Control- Baseline Events	Relative Risk (95% CI)
***Mortality*** **(*****Intrapartum-related Neonatal Deaths*****)** ***: Moderate outcome specific mortality***								
3 [2,38,44]	Before-and-after	Low quality	No evidence of heterogeneity (P=0.5)	Facility settings (ranging primary to tertiary care level), LIC-MIC	Advanced NRP in 2 studies, WHO Basic ENC in another	360*	185	0.70 (0.59, 0.84)^a^

***Mortality*** **(*****Early Neonatal Deaths*****)** ***: Moderate outcome specific mortality***								
3 [3,38,44]	Before-and-after	Low quality	Strong evidence of heterogenity (P=0.002)	Facility settings (ranging primary to tertiary care level), LIC-MIC	Advanced NRP in 2 studies, WHO Basic ENC in another	454*	458	0.62 (0.41, 0.94)^b^

***Morbidity*** **(*****Hypoxic Ischemic Encephalopathy*****)** ***: Low outcome specific morbidity***								
1 [2]	Before-and-after	Low quality	NA	Only 1 study, tertiary care hospital	Advanced NRP	128*	21	1.68 (1.06, 2.66)^c^

a) MH pooled RR; b) D & L pooled RR random effect meta-analysis; c) Directly calculated from study results

* Note numbers of events in post-intervention period are based on longer duration of observation period than baseline

### Intervention descriptions in identified studies

The content and context of the resuscitation training for all facility studies are shown in Tables 2 and 3. Some studies evaluated neonatal resuscitation training as part of a comprehensive perinatal [33-36] or obstetric care program[37], and these evaluations were excluded. In the First Breath study, basic neonatal resuscitation was taught in the first phase as part of an essential newborn care package including bag mask ventilation, then followed by a more in-depth training using elements of the American Academy of Pediatrics Neonatal Resuscitation Program, including immediate assessment and stimulation, bag-mask ventilation and chest compressions [38,39]. Several studies implemented full advanced neonatal resuscitation (American Academy of Pediatrics Neonatal Resuscitation Program [2,3,40-43], French Bulgarian [44], ABCDE [45], or UK resuscitation council training[46]). However, advanced procedures are rarely used (i.e. chest compressions or medications required in < 0.1% of births [11]), the approaches are similar in content, and the additional benefit is likely to be small in low-resource settings. Thus, studies of basic and basic with advanced neonatal resuscitation were combined as long as they had comparable study design and outcome measures.

Several training programs required written and/or clinical practical exam to ensure trainee competency (AAP NRP, UK resuscitation council). Refresher training was conducted in some studies to promote skill maintenance, and is shown in Tables 2 and 3 if reported by investigators.

### Outcomes reported in identified studies

The case definitions for intrapartum-related neonatal deaths (“birth asphyxia”) and preterm mortality varied between studies (Tables 2 and 3). “Asphyxia” mortality was reported in six facility studies [2,3,38-41,44], and was considered in three studies to correspond to term intrapartum-related neonatal mortality [2,38,44]. Among these three studies which were included in the meta-analysis, the sources of cause-of-death data were hospital records in the Indian study [2,3], the National Health Information Centre in the Bulgarian study[44], and a prospective research tracking system with midwives trained in assigning cause-of-death in Zambia[38,39]. The Indian and Bulgarian studies used standard ICD rules to assign a single underlying cause of death. The Zambian study did not use a standard hierarchy to assign single cause of death, and some preterm deaths were possibly assigned to asphyxia. Neonatal mortality due to complications of prematurity was reported separately in the same three studies [2,38,44].  The Bulgarian study [44] used ICD-9 coding to assign cause of death (Immaturity-related or Respiratory Distress Syndrome). The Indian study also used ICD cause of death rules, however required birthweight <1000 with complications of prematurity [2]. The Zambian study used gestational age or weight cutoff (<1500g or <37 weeks) [38,39].

### Meta analyses performed and Delphi panel estimates

We performed meta analyses to summarize the results of studies of neonatal resuscitation training as an isolated intervention with comparable study design for the following outcomes: mortality from intrapartum-related events (n=3 studies), or all-cause early neonatal mortality (n=3) (given that the majority of deaths from term intrapartum events and early preterm deaths occur in the first week of life [47,48]).

The quality of individual studies included in the meta-analyses was assessed by adapted WHO GRADE criteria (Additional file 1) and considered low for cause-specific mortality, although all were set in low-middle income countries and generalizable to the setting of interest. The main limitation was the before-and-after study design, lacking a concurrent control group, and hence the inability to isolate the effect of resuscitation training alone from other changes at the health facilities during the time period, such as improved intrapartum monitoring or post-resuscitation management. Furthermore, the pre-intervention standard of care was not clearly described in several studies and may have differed between facilities, although in all cases presumably included some aspects of immediate newborn assessment and stimulation. The intervention in some cases may have been broader than basic resuscitation alone. An additional limitation of the Zambian study was high rate of loss to follow-up at 7 days (38% pre-intervention and 25% post-intervention). However, this may not have a major effect given that the majority (>70%) of intrapartum-related neonatal deaths occur during the first day of life [47,48], and post-hoc imputations of missing data using maternal and infant characteristics suggest larger magnitude reductions in ENMR after training [38,39].

We excluded 12 studies from the meta-analysis [33-37,40-43,45,46,49].  The Zhu study was excluded as it only reported case fatality ratios for resuscitated newborns, without reporting the total number of births during the observation period [45]. The Tholpadi study was excluded due to the lack of consistent case definitions before and after the intervention [40]. The Draycott, Jeffery, Mufti, Sen, and Berglund studies were comprehensive perinatal training programs that included multiple interventions and did not report intrapartum-related mortality [33-35,37]. The Opiyo study was excluded as only all cause-neonatal mortality was reported[46]. The Wang study was excluded as the primary outcome was immediate death among those with Apgar score <7 in the delivery room, which does not capture all intrapartum-related neonatal deaths nor distinguish deaths due to preterm or other complications. The principal investigators of the study were contacted to try to obtain early neonatal mortality data, but this was not available [41]. The Boo study was not included in the meta-analysis as this ecological study spanned 8 years, the coverage of the intervention was unclear and unequally distributed by state, and intrapartum-related outcomes were not reported [43]. The O’Hare and Duran data were excluded as only deaths among those admitted to the Neonatal Intensive Care Unit were reported [42,49].

#### 1) Basic neonatal resuscitation effect on intrapartum-related term neonatal deaths (“Birth asphyxia”) in facilities

**Figure 3:**
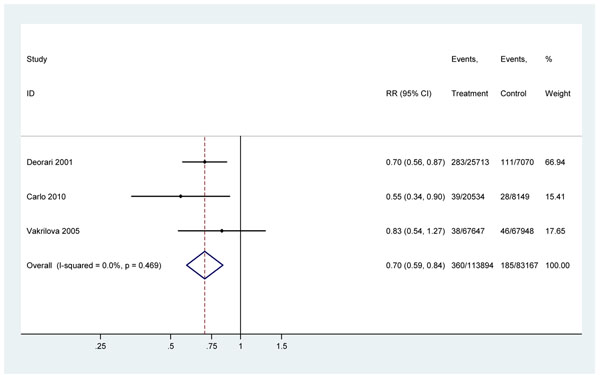
Meta-analysis of before-and-after hospital-based studies examining the effect of additional neonatal resuscitation training on deaths among babies “not breathing at birth”

In this meta-analysis of three studies [2,38,44], training in neonatal resuscitation in the facility setting was associated with a 30% reduction in intrapartum-related mortality (RR=0.70, 95% CI 0.59-0.84) (Figure 3). The direction of effect was protective in all studies, and while effect estimates appeared slightly greater in the higher mortality settings (India, asphyxia-specific mortality rate [ASMR] = 15.7/1000; Zambia, ASMR = 3.4/1000) than in Bulgaria, an upper-middle income country, with relatively low mortality (baseline NMR 7.8, ASMR 0.7/1000), there was not strong evidence of heterogeneity of mortality effect between studies (P=0.47). Given the consistency of the data and generalizability to low-middle income countries, the overall grade of evidence for the effect on intrapartum-related mortality was upgraded to moderate.

#### 2) Basic neonatal resuscitation effect on neonatal deaths due to direct complications of preterm birth in facilities

The same three studies [2,38,44] reported the impact of resuscitation on preterm mortality. However, the study definitions of preterm mortality were heterogenous between studies (Tables 2 and 3) and in 2 studies a very low birth weight cutoff was used [2,38] that would have excluded moderately preterm infants who would be most likely to be saved by basic resuscitation without ongoing intensive care. Thus the study data was not pooled in a meta-analysis. Given the strong biologic plausibility (ie. stimulation, thermoregulation, and positive pressure ventilation at birth may prevent hypoxia and hypothermia, particularly in moderate preterm infants), in combination with the low quality of the evidence, further expert opinion was sought. In the Delphi process, basic neonatal resuscitation was estimated to reduce preterm mortality by about 10% in addition to immediate assessment and stimulation (median opinion 10%, Range 4-30%, IQR 10-20%) (table 5).

**Table 5:** LiST estimates for the effectiveness of immediate stimulation, and of basic neonatal resuscitation on cause-specific neonatal mortality


**Cause of death to act on**	**Newborn assessment and stimulation**	**Basic resuscitation in the community**	**Basic resuscitation in facility**

		**Effect (additional to assessment and stimulation)**	**Effect (additional to assessment and stimulation)**

Intrapartum-related neonatal deaths	DELPHI Median 10% (IQR: 5-15%) (Range: 0-25%)	DELPHI Median 20% (IQR: 15-25%) (Range: 10-50%)	META-ANALYSIS (Figure 2) 30% (95% CI: 16 - 41%)

Neonatal deaths due to complications of preterm birth	DELPHI Median 10% (IQR: 5-10%) (Range: 0-20%)	DELPHI Median 5% (IQR: 5-10%) (Range: 1-40%)	DELPHI Median 10% (IQR:10-20%) (4-30%)

Delphi Expert Opinion estimates based on median answer from Panel of 18 members representing the following:

1) WHO Regions: Americas (n=6); Southeast Asia (n=4); Eastern Mediterranean (n=2); Africa (n=4); Europe (n=2)

2) Specialties: Neonatology (n=7); General Pediatrics (n=11); Pediatric Infectious Disease (n=1)

#### 3) Neonatal resuscitation effect on early neonatal deaths (within 7 days) in facilities

**Figure 4:**
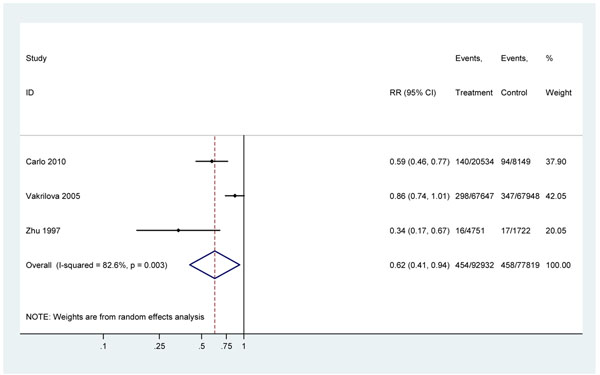
Meta-analysis of before-and-after hospital-based studies of neonatal resuscitation training on early neonatal mortality (all cause)

Almost all (98%) intrapartum-related deaths occur in the first week of life, thus, early neonatal mortality may be a useful proxy measure [47,48]. Three studies were included [3,38,44] in a meta-analysis which suggested that neonatal resuscitation training in the facility setting (2 advanced [3,38,44], 1 basic [38]) was associated with a 38% reduction in early neonatal mortality (RR=0.62, 95% CI 0.41-0.94) (Figure 4). There was evidence of heterogeneity between studies (P=0.003) with a smaller effect observed in the Bulgarian study which had a lower baseline early neonatal mortality rate (ENMR) (5.1/1000) than in the Chinese (9.9/1000) and Zambian studies (11.5/1000).

#### **Evidence for mortality impact of neonatal resuscitation in community settings**

We identified eight studies of neonatal resuscitation training in community-settings that reported mortality outcomes: two cluster-randomized trials (RCT), two quasi-experimental studies, three observational before-after studies and one study with two components, a before-after study followed by a cluster RCT. A detailed description of the studies and their results is shown in Table 6. Preliminary results were available from one cRCT of TBAs undertaking resuscitation in Bangladesh [73], however detailed data was not yet available (M Ellis, personal communication).

**Table 6:** Observational, quasi-experimental, and cluster randomized trials of community-based neonatal resuscitation

Author	Country	Study design	Intervention definition	Simultaneous Interventions	Intervention Coverage	Outcomes: Definition	Preterm vs. Intrapartum Death	N (Births) A = control B = comparison	Effect Size RR/OR (95% CI)
Pratinidhi et al, 1985[50]	Pune, India	Before-and-after	CHW training in basic resuscitation with mouth to mouth	Management of low birth weight, preterm, feeding, illness, cord cutting, feeding, nutrition;	80% of home births received CHW care; 75% of births at home	1) NMR; 2) PMR	Not stated	A) 1444; B) 1546	1) RR 0.75 2) RR 0.98

Daga SR et al, 1991[51]	Maharashtra, India	Before-and-after, no control	TBA training in basic resuscitation with mouth-to-mouth breathing	Management of low birth weight, hypothermia; transport and referral of high risk babies to hospital	TBAs attended 90% of deliveries	1) NMR; 2) PMR; 3) SBR	Not stated	A) 321; B) 660	1) RR 0.59 (0.32-1.09); 2) RR 0.39 (0.21-0.69); 3) RR 0.49 (0.16, 1.50)

Kumar R et a; 1998[55]	Haryana, India	Quasi-experimental	Advanced TBA training modern resuscitation with bag mask ventilation and mucus extractor	NS	TBAs delivered 92% of babies at home;	1) Asphyxia mortality (Verbal Autopsy); 2) PMR	Combined "not breathing"	A) 964; B) 884	1) RR 0.30 (0.11-0.81) 2) RR 0.82 (0.56-1.19)

Bang AT et al 2005[5,72]	Gadichiroli, India	Quasi-experimental	1) 1996-1999: CHW+TBA attend deliveries together, basic resuscitation with tube-mask; 2) 1999-2003: Bag mask. Refresher training every 2 months.	Community treatment of suspected neonatal sepsis, essential newborn care	VHWs attended 84% of deliveries	1) Asphyxia mortality (Verbal autopsy) 2) NMR 3) PMR 4) SBR 5) ENMR	Combined "not breathing" [Failure to breathe at 1, 5 min]	Before-after comparison A) 763 (95-6); B) 5510 (96-03) QE comparison A)1108 B) 979	1) RR 0.35 (0.15-0.78)^a ^ 2) RR 0.41 (0.26-0.66)^b^ 3) RR 0.50 (0.35-0.71)^b^ 4) RR 0.58 (0.36-0.93)^b^ 5) RR 0.44 (0.27-0.73)^b^

Ariawan I, et al 2006[54]	Cirebon, Indonesia	Before-and-after, no control	Community mid-wife training in resuscitation with tube-mask, refresher training 3, 6, 9 month and VCD refresher video; training in "post-resuscitation" care	Not stated	60% of asphyxia cases managed by midwives; uncertain coverage rate	1) Asphyxia mortality (Verbal autopsy); 2) NMR; 3) SBR	Not stated	A) est 44,000; B) est 44,000	1) RR 0.39 (0.31-0.48) 2) RR 0.60 (0.53-0.68) 3) RR 0.39 (0.31-0.48)

Carlo W et al 2010[52]	Argentina, DR Congo, Guatemala, India, Pakistan, Zambia	Before-and-after ENC; cluster RCT for NRP training	Training of community birth attendants (TBAs, nurses, midwives, and physicians) in WHO Essential Newborn Care, including basic resuscitation with bag-mask ventilation	Clean delivery, thermal protection, breastfeeding, kangaroo care	78% of births attended by community birth attendant after ENC training	1) PMR 2) SBR 3) ENMR	BW < 1500 g excluded	A) 22,626; B) 35,017	1) RR 0.85 (0.70-1.02) 2) RR 0.69 (0.54-0.88) 3) RR 0.99 (0.81-1.22)

Gill C et al 2011[53]	Zambia	Cluster RCT	TBA Training in modified neonatal resuscitation program (NRP) w/facemask; competence assessments with refresher trainings every 3-4 mos.	Thermal care, Facilitated referral for presumptive neonatal sepsis (amoxicillin and referral)	Undetermined	1) NMR 2) Day 1 mortality 3) Asphyxia NMR (Verbal autopsy) 4)PMR	Single cause assigned by VA "asphyxia" or "preterm"	A) 1920 B) 1517	1) aRR 0.55, (0.33-0.90) 2) aRR 0.40, (0.19-.83) 3) aRR 0.37 (0.17-0.81) 4) aRR 0.72 (0.51-1.00)

Azad K et al 2011 [73]	Bangladesh	Cluster RCT, factorial design	Intervention arm: TBATraining neonatal resuscitation with bag-valve mask, with subsequent retraining; Control: TBA Training in mouth-to-mouth resuscitation	Intervention and control: Clean delivery, danger signs, emergency preparedness, facility referral. Women's participatory groups in half of clusters	Intervention Coverage: 22% of home deliveries attended by trained TBA; Control 19% by trained TBA	1) ENMR	Not stated	A) 13195 B) 12519	1) 0.95 (0.75-1.21)

a Before-after comparison period 1995-6 versus 1996-2003

b Calculated from data presented in paper for year 3 of intervention (1997-1998) comparing experimental vs. control areas[72]

### Intervention descriptions in identified studies

In the community-based studies, basic neonatal resuscitation was typically implemented as part of comprehensive newborn care packages, often including management of low birthweight babies, hypothermia, and neonatal infections [5,50-53]. In one cRCT [73], in half the clusters participatory women's groups were also implemented.  Ventilation was provided mouth-to-mouth [50,51][73], or by tube-and-mask [5,53,54] or bag-and-mask devices [5,52][73]. The providers ranged from traditional birth attendants [5,51-53,55] to community midwives [38,52,54] to nurses and physicians [52].  In the First Breath study, bag-mask resuscitation training was a component of the essential newborn case (ENC) package [52].

### Outcome definitions in identified studies

When available, cause-of-death was attributed based on verbal autopsy. In most cases, “birth asphyxia” was based primarily on the clinical symptom of “not breathing at birth” and did not exclude preterm infants with respiratory depression; although the First Breath study excluded infants weighing <1500 g [52] and the Lunesp study provided preterm as a separate cause of death[53] .

### Study quality and Delphi panel estimates

The individual study quality for cause-specific mortality effect ranged from very low to moderate; the interventions implemented and case definitions used were heterogeneous. The cluster-randomized component of the First Breath study was excluded as the comparison was between two different training programs of neonatal resuscitation, both including ventilation with bag-and-mask; thus only the before-after essential newborn care training data was considered here [52]. The Lunesp cRCT was rated as moderate quality for the purpose of this review, given the concurrent interventions and hence difficulty separating the effect of resuscitation from sepsis management [53]. Only preliminary results from the Bangladesh cRCT were available [73], the level of evidence may be considered moderate for this review given the lack of cause-specific mortality data and low coverage of the intervention (~20% of deliveries).  Two studies were quasi-experimental with non-random allocation of the intervention and considered to provide low to moderate quality evidence [5,55]. Four other studies were before-and-after studies [50-52,54], providing very low to low quality evidence by GRADE criteria.

Because of substantial heterogeneity in the interventions implemented, the inability to isolate the effect of resuscitation training in community newborn care packages, differences in study design, and the lack of consistent outcomes definitions separating neonatal deaths due to term-intrapartum events vs. preterm birth, no meta-analysis was performed using the community data and the data is summarized.

#### 1) Basic neonatal resuscitation effect on all cause mortality in community based studies

Five studies reported the intervention package effect on all cause perinatal mortality. Three studies reported a 28-61% reduction in PMR [5,51,53], whereas three studies failed to demonstrate a significant effect (RR 0.98, CI not reported[50]; RR 0.82, 95% CI 0.56-1.19[55]; RR 0.85, 95% CI 0.70-1.02[52]). In the First Breath study, however, a sub-analysis of deliveries attended by birth attendants reported a reduction in PMR after vs. before training (RR 0.81, 95% CI 0.68-0.97) [52]. Four studies reported reductions in all cause neonatal mortality ranging from 25-59% [5,50,53,54] and one study failed to demonstrate a significant effect (RR 0.59, 95% CI 0.32-1.09)[51]. Early neonatal mortality was reduced 42% in the Lunesp cRCT (aRR 0.58, 95% CI 0.38-0.89) [53]; however no effect on ENMR was observed in the First Breath before-after ENC comparison (RR0.99, 95% CI 0.81-1.22), most likely due to the large reported reduction of stillbirths, although interpretation may be complicated by misclassification between stillbirths and early neonatal deaths, which is an issue even in high resource settings and is common where routine heart rate assessment at birth is limited [52].  In the Bangladesh cRCT, there was no significant effect on ENMR of bag-mask training of TBAs compared to mouth-to-mouth resuscitation (RR 0.95, 95% CI 0.75-1.21).

#### 2) Basic neonatal resuscitation effect on intrapartum-related neonatal deaths in community-based studies

“Asphyxia” specific mortality was reported for four studies, with the effect ranging from 61-70% reduction [5,53-55]. However, the definition used in three studies was “not breathing at birth” and hence included deaths in preterm infants; only one study distinguished preterm deaths [53]. Sepsis management with antibiotics was a co-intervention in 2 studies [5,53] and study designs were heterogeneous (1 cRCT, 2 quasi-experimental, 1 before-after), thus the data was not pooled. A Delphi expert process was conducted (table 5). Basic neonatal resuscitation was estimated to reduce term intrapartum-related mortality in the community by 20%, in addition to assessment and stimulation (median opinion 20%, Range 10-50%, IQR 15-25%).

#### 3) Basic neonatal resuscitation effect on neonatal deaths due to preterm birth complications in community-based studies

No studies were identified that met criteria for intervention and outcome definitions. The Lunesp study reported no significant reduction in mortality attributed to preterm birth [53]. Given the biologic plausibility, expert opinion was also sought. The Delphi process estimated a 5% reduction, in addition to assessment and stimulation (Range 1-40%, IQR 5-10%) in neonatal deaths due to neonatal resuscitation with positive pressure ventilation in the community (table 5).

#### 4) Basic neonatal resuscitation effect on stillbirths in community-based studies

In the First Breath study, the stillbirth rate was reduced by 31% after the intervention, and in the SEARCH study, the fresh stillbirth rate was 32% lower during the period of bag-mask compared to tube-mask resuscitation (p< 0.09). In the Lunesp study, there was no significant effect of the intervention on stillbirth rate [53].

### Evidence for impact of immediate newborn assessment and stimulation

We identified no studies which reported mortality outcomes for newborn assessment and stimulation alone in the community, or in facilities; therefore, an expert Delphi process was undertaken.

#### 1) Intrapartum-related neonatal deaths

The median opinion was for a 10% reduction (Range 0-25%, IQR 5-15%) in term intrapartum-related deaths with immediate newborn assessment and stimulation alone.

#### 2) Neonatal deaths due to direct complications of preterm birth

The median opinion was for a 10% reduction (Range 0-20%, IQR 5-10%) in preterm deaths following immediate newborn assessment and stimulation alone.

### Mortality effect, combining stimulation and basic resuscitation

The total effect of basic resuscitation is estimated as the effect of newborn assessment and stimulation, and the additional effect of basic resuscitation on the remaining deaths, after subtracting the lives saved from initial newborn assessment and stimulation (table 5). In the meta-analysis, the additional effect of basic resuscitation included studies where training with bag-and-mask was implemented on top of existing basic newborn care. In the Delphi, the effect of basic resuscitation was incremental to newborn assessment and stimulation. For example, if there are 1000 intrapartum related deaths in the absence of any care, introducing newborn assessment and stimulation for all children would be expected to prevent 10% of these deaths (=100), leaving 900 deaths still occurring. Adding basic resuscitation in the community to newborn assessment and stimulation would prevent 20% of these remaining deaths (=180). Thus, the total number of deaths prevented would be 280 (=28%). In the LiST software, assessment and stimulation is included with skilled attendance for facility birth and the basic resuscitation is a separate additional option.

### Summary of the results and the quality of evidence

The LiST mortality effects for the two interventions (immediate newborn assessment and stimulation, and basic neonatal resuscitation) on the two causal categories of neonatal death (term intrapartum-related and preterm birth complications) are summarized in table 7, along with evaluations of quality of evidence, or expert opinion, and limitations of the data. The overall level of evidence for facility based neonatal resuscitation impact on term intrapartum related mortality was based on a meta-analysis of 3 studies and was rated as moderate, while all the remaining estimates were based on Delphi expert consensus and the quality of the evidence was rated very low.

**Table 7:** Cause specific mortality effects and GRADE estimate for the effect of newborn resuscitation


**Effect on intrapartum-related neonatal deaths** (“birth asphyxia”)

***Cause specific effect*** Immediate newborn assessment, drying, and stimulation 10% (Range 0-25%, IQR 5-15%) Basic neonatal resuscitation (facility) 30% (95% CI: 16 - 41%) Basic neonatal resuscitation (community) 20 % (Range 10-50%, IQR 15-25%) (*note that the resuscitation effect is in addition to immediate assessment, drying, and stimulation) ***Quality of input evidence:*** Basic neonatal resuscitation (facility) - moderate (3 low quality before-and-after studies, upgraded for consistency) Immediate newborn assessment, drying, and stimulation - very low (based on Delphi) Basic neonatal resuscitation (community) - very low (based on Delphi) ***Proximity of the data to cause specific mortality effect:*** Moderate (cause specific mortality but lack of consistency in cause-of-death definitions) ***Limitations of the evidence:*** There is a lack of rigorous evaluation particularly for the effect of immediate newborn assessment, drying, and stimulation. Data are compromised by misclassification of live births and intrapartum stillbirths and by inconsistencies in cause-of-death attribution between term intrapartum-related neonatal deaths and preterm complications especially if a clinical case definition of “not breathing at birth” (“birth asphyxia”) is applied which includes both categories. ***Possible adverse effects:*** Babies who survive despite severe brain injury may have long term impairments. There is a dearth of data on long term outcomes from low and middle income settings.

**Effect on neonatal deaths due to preterm direct complications**

***Cause specific effect*** Immediate newborn assessment, drying, and stimulation 10% (Range 0-20%, IQR 5-15%) Basic neonatal resuscitation (facility) 10% (Range 4-30%, IQR 10-20%) Basic neonatal resuscitation (community) 5% (Range 1-40%, IQR5-10%) (*note that the resuscitation effect is in addition to immediate assessment, drying, and stimulation) ***Quality of input evidence:*** Very low (all based on Delphi) ***Limitations of the evidence:*** As discussed above. ***Possible adverse effects:*** As discussed above.

## Discussion

Despite the wide acceptance of neonatal resuscitation as a standard of care, there is limited evidence of its impact on neonatal outcomes, in part due to the ethical challenges of undertaking individually randomized RCTs. To our knowledge, this is the first systematic review, meta-analysis and expert panel convened to provide estimates of the reduction in neonatal mortality that could be achieved through neonatal resuscitation training. Immediate assessment and stimulation of the newborn is more feasible without equipment or skilled workers. Our expert panel estimated that this simple action could reduce both term intrapartum-related (ie. “birth asphyxia”) and preterm mortality by 10%. Our meta-analysis suggests that neonatal resuscitation training in facilities was associated with an additional 30% reduction in intrapartum-related neonatal mortality. Studies have not consistently assessed the effects on preterm deaths, and there is no high or moderate quality evidence addressing this; expert opinion estimated a 10% reduction in prematurity-related neonatal deaths following resuscitation in health facilities. Current evidence for neonatal resuscitation in community settings is heterogeneous, and experts estimated a 20% reduction in term intrapartum-related deaths and 5% reduction in deaths attributed to preterm birth for community-based resuscitation either with a midwife alone at home or a TBA.

Simple immediate newborn assessment and warming, drying and tactile stimulation is the first step of neonatal resuscitation and was estimated by experts to result in a small (10%) reduction in intrapartum-related (“birth asphyxia”) and preterm deaths. In resource limited settings, these simple initial steps are feasible to be performed by a family member or primary healthcare provider with minimal skills – for example, rubbing the baby dry with a cloth– and might save lives, but this is expected to have limited effect. Observational studies suggest that between 6-42% of newborns who do not breathe at birth require ventilation [2,54,55], indicating that the majority of non-breathing babies may respond to simple steps alone. Although the anticipated mortality impact is low, the cost is also likely to be low as no equipment is required.

Our meta-analyses evaluating the impact of facility-based neonatal resuscitation training included low quality before-after studies, but at least comparable in intervention and outcome definitions for intrapartum-related and early neonatal mortality. Consistent effect sizes were observed for intrapartum-related mortality and all cause early neonatal mortality. The China NRP study [41] was excluded but it is notable that the reduction in labour room mortality for term babies (33%) was of similar magnitude. It is disappointing that the majority of the 16 facility studies identified did not meet inclusion criteria. However, given mortality effect consistency across the studies and generalizability to low-middle income countries, applying adapted GRADE criteria the evidence level was moderate (table 7). For all included studies, the comparison groups involved some pre-training management of the non-breathing baby, thus, these estimates reflect the impact of additional training for resuscitation, incremental to immediate newborn assessment and stimulation. Implementing basic neonatal resuscitation in a setting where no simple immediate newborn care is in place, such as peripheral maternity clinics, may have a greater effect. On the other hand, some of the effect may have been due to improved post-resuscitation care in two of the studies [2,44]. While some data was available on the impact of facility-based resuscitation on preterm mortality, the data was too heterogeneous to pool. However, there is strong biologic plausibility that resuscitation may reduce mortality in moderate-late preterms who require minimal assistance with positive-pressure ventilation to initiate breathing, without requiring ongoing assisted ventilation; experts estimated a10% effect at facility level.

The impact of resuscitation training may be greater in higher mortality settings where obstetric care is more limited. In Bulgaria, an upper-middle income country where the baseline intrapartum-related mortality was relatively low, the estimated effect was smaller (16%) than in higher mortality settings such as Zambia and India, where neonatal resuscitation training was associated with a 30-43% reduction in intrapartum-related mortality. In settings with high coverage of high quality intrapartum management, the majority of term infants who die from intrapartum-related causes may be severely asphyxiated infants who require interventions beyond neonatal resuscitation alone, such as ongoing ventilation and therapeutic hypothermia.

The evidence for basic resuscitation in community settings was too heterogeneous to combine: study designs varied substantially, resuscitation training was one of numerous interventions in newborn care packages, and the outcome measure of cause-specific mortality differed across studies, often reflecting reduction in other causes of death such as preterm birth and infections. Significant reductions in all-cause neonatal or perinatal mortality were observed in 4 studies, ranging from 25-61% [5,53-55], and reported “asphyxia” specific mortality was reduced in four studies, ranging from 61-70% [5,53-55]. In the multi-center “First Breath” study [52], although no overall impact on PMR was observed, there was a significant 19% PMR reduction for deliveries with trained birth attendants, and a reduction in intrapartum-related morbidity (prevalence of 5 minute Apgar scores <4 and abnormal neurologic exams at 7 days). On the other hand, preliminary results from a cRCT in Bangladesh failed to demonstrate a reduction in ENMR with the additional training of TBAs in bag-mask resuscitation beyond immediate care and mouth-to-mouth resuscitation.  Although it was not possible to derive a cause-specific mortality estimate from existing evidence, our expert panel agreed on the presence of an effect (20% for intrapartum-related mortality, 5% for preterm mortality), albeit slightly smaller than for facility based resuscitation, reflecting the additional challenges in implementation in such contexts, with a single provider and variable cadres. There is a need for consistency in future studies with respect to intervention content, study design, outcome measurement and definitions in order to more precisely evaluate the potential impact of resuscitation training at community level.

Important programmatic considerations for resuscitation training in resource limited settings include the benefit of teaching advanced procedures, provider competency, and skill maintenance. Two of the studies in our meta-analysis included some aspects of advanced neonatal resuscitation; however, advanced procedures are more complex to teach (i.e. chest compressions, intubation, or medications) and are required for ~2% of all babies who do not breathe at birth[2,56], and fewer than 1% of all babies born[6,11]. Basic neonatal resuscitation is sufficient for most babies who would be saved by resuscitation in low-middle income settings, and the additional benefit of advanced procedures is likely to be low. For the purposes of this LiST estimate, the effect of facility based neonatal resuscitation was assumed to be achievable with basic neonatal resuscitation, which is the clear priority for rapid scale up in facilities in low and middle income countries, given feasibility, skills required, and equipment costs. Furthermore, training programs should emphasize routine assessment of provider knowledge, competency and skill maintenance. Provider knowledge and performance skills to conduct resuscitation decline significantly over time[57]. Regular refresher training programs, practice drills, and DVD videos of resuscitation are methods of ensuring skill maintenance and program effectiveness[1,58] .

A reduction in stillbirth rate has been observed in 2 community-based studies, after training programs including bag-mask resuscitation [5,52]. A live newborn with severe neonatal depression is difficult to distinguish from a stillborn, and there is the potential for misclassification in low-resource settings where newborns are not typically assessed for signs of life at birth (particularly heart rate) [59,60]. In addition to reducing misclassification, training in neonatal assessment and resuscitation may also increase survival in apparently stillborn infants (Apgar score assessed as 0 at 1 minute). Among apparently stillbirth infants who were resuscitated, case fatality ranges between 16-65% in high income settings [61-63], with major intensive care support, and long term outcomes that are significantly worse than for resuscitated babies who did have a heart rate detected [64]. These findings emphasize the need to accurately count stillbirths and assess long term outcomes to capture the full impact of obstetric and immediate newborn care interventions [65,66].

Consistent case definitions are required for comparable population-level surveillance of disease burden and for evaluation of intervention effectiveness. A survey of policy makers revealed that “confusing terminology” and “lack of valid measurement indicators at the community level” were key barriers to obtaining the necessary information to make policy decisions[19]. Recent advances have been made in case definitions and verbal autopsy hierarchies to distinguish intrapartum-related events in term or almost term babies from preterm babies, although the issue of distinguishing growth restricted infants remains a challenge and is especially important in South Asia. Consistent use of such verbal autopsy tools, and more importantly the hierarchies, is critical [67]. This review emphasizes the need to minimize misclassification of live births as stillbirths, and to apply standardized definitions for intrapartum-related neonatal deaths, as opposed to clinical definitions such as “birth asphyxia.” Definitions and measurement varied across studies and between facility and community/home-based studies. Even in facility settings, the few studies that reported preterm mortality used inconsistent birth weight and gestational age cut-offs. There is a marked lack of data regarding effect of resuscitation on preterm babies. The long-term developmental outcomes following resuscitation also require further research. Particularly in low-middle resource settings, where health systems and families have limited resources to care for survivors with chronic disability, there is a dearth of comparable long term developmental outcome data (ACL, personal communication for CHERG/GBD neonatal encephalopathy estimates group).

This review has important implications for the scale up of neonatal resuscitation. The immediate opportunity is for facility based resuscitation. Even in facilities, equipment is lacking and few providers are trained in neonatal resuscitation. In 6 African national service provision assessments (DHS Macro), between 2-12% of delivery staff had been trained in neonatal resuscitation and fewer than one quarter of hospitals had newborn bag-masks available [1]. Given these challenges, achieving high coverage with basic neonatal resuscitation should be prioritized, as advanced resuscitation is infrequently required and may have limited additional mortality impact in low-resource settings. Establishing resuscitation training for pre-service education of midwives, doctors and nurses who provide newborn care is a crucial step. Recent advances in simpler training and robust, low cost equipment hold great promise for rapid scale up at much lower cost [68]. Furthermore, for the 60 million births a year outside facilities, while implementing basic neonatal resuscitation at the community level is controversial, there may be a role in some high-mortality settings where most births occur at home, skilled attendance is not achievable in the foreseeable future, alternative cadres already attend the majority of deliveries, and the case load per attendant is high enough to justify the training, equipment inputs and skill maintenance.

## Conclusion	

There is evidence from facility-based studies in low and middle-income countries that neonatal resuscitation training reduces neonatal mortality from intrapartum-related events (ie. “birth asphyxia”) by 30%, potentially saving 93,700 each year just by addressing missed opportunities for current facility births, and up to 192,000 babies at 90% coverage [69], only considering the effect on intrapartum-related neonatal deaths. In order to achieve maximal reduction in intrapartum-related neonatal deaths, preterm birth and intrapartum stillbirths, effective obstetric care remains the most important intervention [70,71] and this should be complemented with immediate newborn care and resuscitation. There is increasing investment in obstetric care, yet to be matched by effective implementation, scale-up, and sustainability of immediate newborn care and basic neonatal resuscitation.

In the community, immediate simple care at birth is feasible, although estimated by experts to be low impact (10% on preterm and on intrapartum related neonatal deaths). Community-based neonatal resuscitation may reduce all-cause neonatal and perinatal mortality, but data is heterogeneous to presently estimate an effect size from the evidence. Future studies should attempt to address limitations identified here particularly in terms of intervention definitions, design, comparison groups, outcome definitions and misclassification of stillbirths and neonatal deaths.

While the quality of evidence for stimulation at birth and for neonatal resuscitation is low, partly because they are considered standard of care, there is sufficient and consistent evidence of impact. Yet such basic care remains a rarity especially for the world’s 60 million home births. Simplified training programs, and robust, low cost equipment are now available. Every baby born alive has the right to breathe at birth and to solutions helping those who do not breathe – the question remains if this right will be systematically advanced in policies and programs or will be left to chance depending on where a baby is born.

## List of abbreviations used

WHO: World Health Organization; CHERG: Child Health Epidemiology Reference Group; GBD: Global Burden of Disease; IQR: Inter quartile range; LiST: Lives Saved Tool; PMR: perinatal mortality rate; NMR=neonatal mortality rate; ENMR: early neonatal mortality rate

## Competing interests

The authors declare that they have no competing interests.

## Authors’ contributions

ACL undertook the searches and abstraction with JL. ACL, SC, and JL undertook the meta-analyses. ACL, JL, and SW designed and implemented the Delphi process. All authors contributed to the manuscript. The authors declare no conflict of interest.

## Funding

This work was supported by the Bill & Melinda Gates Foundation through a grant to US Fund for UNICEF for work on LiST and to the Saving Newborn Lives program of Save the Children US.

## Supplementary Material

Additional File 1 This file is an excel file that contains three work sheets. The first sheet has the quality coding of studies used in the meta-analysis, the second sheet has the Grade rating of the evidence and the third sheet has the CHERG rules for defining the effectiveness values. Click here for file

Additional File 2 This file is a word document. It contains the Delpi documents used by the experts in the Delphi process. Click here for file
