# Supplementary material for: Neonatal resuscitation and immediate newborn assessment and stimulation for the prevention of neonatal deaths: a systematic review, meta-analysis and Delphi estimation of mortality effect
Source: BMC Public Health. 2011 Apr 13;11(Suppl 3):S12. doi: 10.1186/1471-2458-11-S3-S12 (PMC3231885; doi:10.1186/1471-2458-11-S3-S12)
Supplement: Additional File 2 — This file is a word document. It contains the Delpi documents used by the experts in the Delphi process. [file 1471-2458-11-S3-S12-S2.docx]

**Additional File 2. Questionnaire for Delphi Expert Consensus**

### Lives Saved Tool (LiST) Expert consensus panel via a Delphi process

Estimates of the effectiveness of neonatal resuscitation on cause specific neonatal mortality

**Background to LiST**

The Lives Saved Tool (LiST) is based on *The Lancet* Child Survival and Neonatal Survival series modelling for lives saved and is now built into a widely accepted demographic software package (Spectrum^TM^). The core of Spectrum is a demographic projection model which projects the population by age and sex. LiST is a module that incorporates recent mortality rates by country and cause-of-death data for newborns and children based on definitions and estimates established by the Child Health Epidemiology Group (CHERG). The LiST tool models the impact of increasing coverage of individual interventions on the reduction of neonatal deaths by specific cause-of-death. To use the LiST tool, the user is given a menu of interventions preset with estimates of current national coverage level from Demographic and Health Surveys, and then sets coverage targets for each intervention by year up to 2015. The increases in coverage are linked to cause-specific mortality effect estimates. The estimates of lives saved are modelled such that neonatal deaths are assigned a single cause and lives cannot be saved twice by linked interventions. A prototype of the tool can be downloaded at: [**h**ttp://www.healthpolicyinitiative.com/index.cfm?id=software&get=Spectrum](http://www.healthpolicyinitiative.com/index.cfm?id=software&get=Spectrum)

The mortality effect estimates used in the tool are based on a consistent literature review process presently being led by CHERG, using an adapted version of the WHO GRADE criteria to evaluate the quality of evidence, and conducting meta-analyses of intervention effect size where appropriate. In cases of insufficient evidence, expert opinion is being sought in order to arrive at effect estimates. The detailed technological basis, cause-specific mortality reviews and effect sizes used in the LiST tool will be published in a peer review journal supplement so that the assumptions and inputs are all in the public domain.

**Background to this Delphi process**

You have been approached to participate in this Delphi process because of your expertise in this topic. We are asking you to answer 5 questions on the following page. We will collate the responses from the group. If there is strong consensus on the first round, a second round will not be necessary but in many cases two or even three rounds are required. To meet the deadline to be included in LiST we need to complete the estimates by late April.

**Specific background regarding the effect estimates on neonatal resuscitation**

Given that it would be unethical to undertake randomized trials of resuscitation compared to no resuscitation, the evidence for resuscitation effect on neonatal mortality is very low. After systematic literature review no studies were identified which examined just the effect of simple stimulation at birth (assessment, drying, wrapping etc). We identified 6 before and after studies of facility-based basic resuscitation. Although low quality, the results are very consistent and a meta-analysis of 3 studies showed a 30% reduction in deaths due to “birth asphyxia” in full term infants. Community-based studies of resuscitation were all part of packages with multiple concurrent interventions , making it difficult to determine the effect of resuscitation alone, and furthermore most community studies used definitions of “not breathing at birth” which included preterm infants

An additional challenge is the definition of “birth asphyxia” varies between trials, and many published trials have measured the effect on deaths amongst babies “not breathing at birth” which is a clinical definition and does not match the epidemiological definitions used for cause of neonatal death in the LiST modeling. Mortality effect of resuscitation is expected on two causes of death (as per the CHERG case definitions):

1) intrapartum-related term neonatal deaths (previously referred to as “birth asphyxia”)

2) preterm birth (gestational age <34 weeks, birth weight <2000g , or specific preterm complications, including respiratory distress syndrome, intraventricular hemorrhage, and necrotizing enterocolitis)

**Expert opinion requested from you**

**Intervention:** We are asking you to estimate the percentage of newborn lives saved by either simple immediate newborn care at birth, including drying and stimulation, or basic neonatal resuscitation with bag and mask, but with no other new interventions at the same time.

**Causes of death to act on**: The estimate refers to percentage reduction in each of the two causes of death expected to be affected by resuscitation. In LiST the data is preloaded for each country with the estimates for these causes of death and our estimated mortality reduction will link to these causes:

1) intrapartum-related term neonatal deaths (previously referred to as “birth asphyxia”)

2) preterm birth (gestational age <34 weeks, birth weight <2000g , or specific preterm complications, including respiratory distress syndrome, intraventricular hemorrhage, and necrotizing enterocolitis)

**Mortality effect:** We request that you provide your best estimate of the % of neonatal deaths that could be prevented for each of these causes if the proposed intervention had been available to all who died from this cause. The comparison group you should imagine is the unattended birth where the newborn is not assessed at all. Note that not all babies who do not breathe at birth will die and not all babies “saved” at the time of the birth by resuscitation will survive, especially in the absence of intensive care – eg a baby with severe intrapartum injury may then die of NE or a baby with extreme preterm birth may die of RDS. Finally, these estimates are for effectiveness in a “typical,” real-life developing country setting as opposed to efficacy under optimal conditions in a research study.

|  | Estimated Impact on Neonatal Mortality Reduction for.. | |
| --- | --- | --- |
| Intervention | Full term asphyxiated neonates (intrapartum-related neonatal deaths) | Preterm neonates with cause-of-death attributed to preterm complications |
| **Simple Immediate Newborn Care** Including, drying, stimulation | 1 = ___% | 2 = ___% |
| **Basic Neonatal resuscitation with Positive Pressure Ventilation**  Including clearing airway/suctioning, head positioning and PPV via bag mask or tube mask, but NO chest compressions, medications or intubation | **30%**  based on a new meta-analysis of 3 studies of facility-based neonatal resuscitation | 3 = ___%  (effect in facility setting) |
| **Basic Neonatal resuscitation with Positive Pressure Ventilation**  Including clearing airway/suctioning, head positioning and PPV via bag mask or tube mask, but NO chest compressions, medications or intubation | 4 = ___%  (effect in home setting) | 5 = ___%  (effect in home setting) |

(1) If there are 100 full term babies who are currently dying from intrapartum-related events (“birth asphyxia”), how many would be revived and survive to 28 days of life with only **simple immediate newborn care at birth**?

(2) If there are 100 babies born who are currently dying of direct complications of preterm birth, how many of those would be revived and survive to 28 days of life by receiving only **simple immediate newborn care at birth**?

(3) If there are 100 babies born who are currently dying of direct complications of preterm birth, how many of those would be revived and survive to 28 days of life by receiving **basic neonatal resuscitation with positive pressure ventilation using a bag and mask** in a hospital facility?

(4) In the scenario of a ‘typical’ home birth in a developing country, if there are 100 full term babies who are currently dying from intrapartum-related events, how many would be revived and survive to 28 days of life with **basic neonatal resuscitation with positive pressure ventilation using a bag and mask**?

(5) In the scenario of a ‘typical’ home birth in a developing country, If there are 100 babies born who are currently dying of direct complications of preterm birth, how many of those would be revived and survive to 28 days of life by receiving **basic neonatal resuscitation with positive pressure ventilation using a bag and mask**?

Your name: ______________________________ Email: ________________________

Affiliation: ______________________________[Leave all blank if you prefer to submit anonymously]
